# Mammal Molar Size Ratios and the Inhibitory Cascade at the Intraspecific Scale

**DOI:** 10.1093/iob/obaa020

**Published:** 2020-07-24

**Authors:** N S Vitek, C C Roseman, J I Bloch

**Affiliations:** 1 Department of Ecology & Evolution, Stony Brook University, 632 Life Sciences Building, Stony Brook, NY 11794-5245, USA; 2 Florida Museum of Natural History and Department of Biology, University of Florida, Gainesville, FL 32611, USA; 3 Department of Evolution, Ecology, and Behavior, University of Illinois, Champaign, IL 61820, USA

## Abstract

Mammalian molar crowns form a module in which measurements of size for individual teeth within a tooth row covary with one another. Molar crown size covariation is proposed to fit the inhibitory cascade model (ICM) or its variant the molar module component (MMC) model, but the inability of the former model to fit across biological scales is a concern in the few cases where it has been tested in Primates. The ICM has thus far failed to explain patterns of intraspecific variation, an intermediate biological scale, even though it explains patterns at both smaller organ-level and larger between-species biological scales. Studies of this topic in a much broader range of taxa are needed, but the properties of a sample appropriate for testing the ICM at the intraspecific level are unclear. Here, we assess intraspecific variation in relative molar sizes of the cotton mouse, *Peromyscus gossypinus*, to further test the ICM and to develop recommendations for appropriate sampling protocols in future intraspecific studies of molar size variation across Mammalia. To develop these recommendations, we model the sensitivity of estimates of molar ratios to sample size and simulate the use of composite molar rows when complete ones are unavailable. Similar to past studies on primates, our results show that intraspecific variance structure of molar ratios within the rodent *P. gossypinus* does not meet predictions of the ICM or MMC. When we extend these analyses to include the MMC, one model does not fit observed patterns of variation better than the other. Standing variation in molar size ratios is relatively constant across mammalian samples containing all three molars. In future studies, analyzing average ratio values will require relatively small minimum sample sizes of two or more complete molar rows. Even composite-based estimates from four or more specimens per tooth position can accurately estimate mean molar ratios. Analyzing variance structure will require relatively large sample sizes of at least 40–50 complete specimens, and composite molar rows cannot accurately reconstruct variance structure of ratios in a sample. Based on these results, we propose guidelines for intraspecific studies of molar size covariation. In particular, we note that the suitability of composite specimens for averaging mean molar ratios is promising for the inclusion of isolated molars and incomplete molar rows from the fossil record in future studies of the evolution of molar modules, as long as variance structure is not a key component of such studies.

The study of mammalian tooth crowns is necessarily the study of interrelated modules that covary because of genetic, developmental, and functional relationships (van Valen 1962; Klingenberg 2008; Grieco et al. 2013). Individual traits on a tooth crown can not only covary with each other, but also with those of other teeth (van Valen 1962; Kangas et al. 2004; Carter and Worthington 2016). In this light, studying phenotypic covariation is a promising path forward to understanding the evolution of this complex set of biological structures (Kurtén 1967; Renaud et al. 2017; Delgado et al. 2019).

Within the mammalian molar module, one of the primary patterns of covariation is the relative sizes of each molar crown (Butler 1939; Kavanagh et al. 2007; Halliday and Goswami 2013; Evans et al. 2016; Hlusko et al. 2016). The inhibitory cascade model (ICM) proposes a developmental and genetic link underlying this suite of traits (Kavanagh et al. 2007). Under the strict form of the ICM as well as proposed modifications to the model, molar crown size proportions are related to the self-regulation of activator and inhibitor molecules influencing the final size of sequentially developing tooth germs, resulting in a mesiodistal trade-off in size (Kavanagh et al. 2007; Young et al. 2015).

One confusing observation in previous studies is that the ICM does not appear to scale neatly with increasing biological scale (Kavanagh et al. 2007; Bernal et al. 2013; Roseman and Delezene 2019). The model explains variation at the intra-individual organ scale in developing tooth germ explants of *Mus musculus*. There, different amounts of exposure to M_1_ inhibitor resulted in different sizes of M_2_ and M_3_ (Kavanagh et al. 2007). The difference between the resulting tooth size ratios between experimental treatments follows the predictions of the ICM (Kavanagh et al. 2007). At a larger clade scale, the ICM can describe differences between species in murid rodents, though it does not always adequately describe variation in other clades (Polly 2007; Labonne et al. 2012; Halliday and Goswami 2013; Carter and Worthington 2016; Couzens et al. 2016). These results imply that at an intermediate within-species or between-population scale relative molar sizes should also follow predictions of the ICM. Studies published thus far analyzing this biological scale in primates do not meet these predictions (Bernal et al. 2013; Roseman and Delezene 2019). However, it is unclear if primates in particular are poor models for studying the ICM or if the ICM fails at this intermediate scale more broadly across Mammalia.

The between-individual, between-population, and between-sister-species biological scales are critical for understanding the evolutionary processes underlying molar variation. Such studies must meet key sampling criteria. To study modern mammals, researchers must make new collections of large numbers of specimens or rely on the prescience of previous collectors for natural history museums to collect adequate samples of multiple populations of single species (Holmes et al. 2016). It is not yet clear what constitutes adequate sample size for intraspecific study of the ICM.

The fossil record is an additional, promising resource with which to study this biological scale because it is the only record capable of sampling the same species over long time scales (10^4^–10^6^ years). It also contains irreplaceable evidence of how this system might be affected by shifts in climate, ecology, and evolution, including intervals of mass extinction and major transitions in the evolution of clades. Mammalian molars make up a large component of the fossil record, but additional challenges have prevented its use for this purpose. Samples of fossils are always time-averaged to some degree, and if too large a span of evolutionary change is averaged into a sample then results may not be valid (Barnosky 1990; Roy et al. 1996). Comparing variance of samples through the coefficient of variation (CV) is the primary method for assessing this possibility, but it is not clear if the rule of thumb that species CV < 10 applies to molar ratio values (Barnosky 1990; Plavcan and Cope 2001). In addition, fossils are usually incomplete and highly fragmented. Finding one complete molar row of a species is uncommon, but possible (Labonne et al. 2012; Halliday and Goswami 2013). Finding a large sample size of complete molar rows of a single species limits study to very common species or extraordinarily well-preserved fossil Lagerstätten (Briggs 2001; Lyson et al. 2019).

The purpose of this study is to characterize additional intraspecific properties of the mean, variance, and covariance of molar ratio phenotypes to facilitate future intraspecific study of the ICM. To achieve this goal, we document standing levels of variation and sensitivity to sample size in order to provide recommendations to future studies in terms of what kinds of samples and measurements might be appropriate for addressing the ICM at this scale. We also assess the amount of error introduced by creating point estimates of molar ratios from a composite of isolated molars. Although such a practice may seem ridiculous from the viewpoint of modern specimens where complete mandibles or hemi-mandibles are the norms rather than the exception, it would greatly benefit studies of the zooarcheological and paleontological records where large samples of complete molar rows of a single species are rare but large samples of isolated teeth are more common. Standard practice in previous paleontological studies has been to exclude samples of isolated molars due to concern of intraspecific variation (Halliday and Goswami 2013). That practice has limited other studies of the ICM in the fossil record in the past (Halliday and Goswami 2013; Schroer and Wood 2015), but the limitation may be more perceived than real. In particular, we compare this practice to the alternative approach of limiting studies to the small number (*N* < 5) of complete molar rows that might be available for any given species in the fossil record (Labonne et al. 2012). Future studies of molar proportions and their relationships to developmental and genetic modules require an evaluation of sample suitability for the research question. To aid in this assessment, we document standing levels of variation in modern populations and species, as well as a test of the assumption that isolated molars may not provide an accurate estimate of population-level phenotype. By characterizing these ratio phenotypes in terms of their intraspecific variation and analyzing the degree to which sampling affects estimated properties of these phenotypes, we aim to help develop best practices for furthering our understanding of how developmental and genetic processes scale with changes in magnitude of evolution. To achieve this goal, we use samples from the published literature as well as a new sample of measurements of the cotton rat, *Peromyscus gossypinus*, which may be more likely to follow predictions of the ICM than the previously studied primates by virtue of its identity as a cricetid rodent. With these samples, we address four questions:

Are particular measurement types better for studying molar size ratios in the ICM, as has been proposed (Hlusko et al. 2016)?
What is the approximate amount of standing variation in molar size ratios at the population level and species level?
How sensitive are estimates of the mean and error to changes in sample size?
Does the use of composite molar rows instead of complete molar rows change the estimated amount of variation or mean ratio for a species or population?

Answers to these questions will help guide future research of covarying morphotypes, such as better understanding how well the ICM functions as a line of least resistance (Schluter 1996; Young et al. 2015). If the model meets the expectations of lines of least resistance, it may serve as a framework for connecting evolutionary observations at a microevolutionary (within species) scale to those at a macroevolutionary (between species or clades) scale. If it does not, then the ICM serves as an equally interesting avenue of research to understand how potentially disparate biological processes operating at different biological scales can produce similar morphological patterns.

## Predictions of ICM

In its most specifically developed form, the ICM predicts the results of a relationship between activator and inhibitor where the effect of activator and inhibitor relative to each other is constant (Kavanagh et al. 2007; Young et al. 2015). That constant is summarized by the ratio of M_2_ crown size relative to that of M_1_  (Kavanagh et al. 2007; Hlusko et al. 2006). Across the molar row, the constant is a simplification of a linear relationship between activator and inhibitor described by the Equation 1+[(*a* − *i*)/*i*](*x* − 1), where *a* = activator, *i* = inhibitor, and *x* = tooth position (Young et al. 2015).

How best to measure tooth size is under some debate (Renvoisé et al. 2009; Hlusko et al. 2016; Gomes Rodrigues et al. 2017). A common, simple way to measure size is the rectangular estimate of crown area based on length and width (Wilson et al. 2012; Bernal et al. 2013; Halliday and Goswami 2013; Schroer and Wood 2015; Asahara et al. 2016; Evans et al. 2016; Roseman and Delezene 2019). More precise, outline-based measurements of two-dimensional crown area reduce the amount of error introduced into analyses to varying degrees (Kavanagh et al. 2007; Renvoisé et al. 2009; Carter and Worthington 2016; Gomes Rodrigues et al. 2017). However, quantifying size in terms of molar area measured in any way may necessarily subject the ICM to pleiotropic genetic influence of body size because of genetic correlations between buccolingual width and body size (Hlusko et al. 2006). A proposed more direct measure of a single genetic module that follows some ICM-like developmental pathways is the molar module component (MMC). The MMC is measured by mesiodistal length, which should follow the same limited sets of relationships among molar sizes as the ICM assuming that the length of the M_2_ is predictable from any given M_1_/M_3_ ratio. This assumption holds for the primates in which it has been studied (Hlusko et al. 2016). If the MMC is a more precise way of capturing the ICM, length measurements should match ICM predictions more closely than area measurements for the same specimens because there should be reduced pleiotropy and therefore lower likelihood of overprinting of other genetic modules (Hlusko et al. 2016; Monson et al. 2019).

The ICM and possibly the MMC are each one specific model among a more general class of ascending-descending (AD) models in which “segments interact in a constant direction … but the magnitude of the effect varies between any given pair of segments,” such that differences in phenotype are compounding and cumulative even if that the degree of compounding is non-linear across the tooth row (Young et al. 2015, 6). Put another way, teeth have shared covariance with a ratcheting effect (Kavanagh et al. 2007; Polly 2007). The space contained in the AD model space is mathematically equivalent to the “ICM-consistent” space of Polly (2007). As such, the AD model still largely applicable to most groups of extant mammals, including metatherians with more than three molars (Polly 2007). It is appealing in that it provides a well-defined morphospace but the biological meaning is less clear than the ICM because the AD model lacks the explicit links to a describable activator-inhibitor ratio that gives the ICM so much predictive power. If a sample fits the AD model but not the ICM, it is difficult to determine whether a nonlinear effect of activator to inhibitor underlies the results, or if additional interactions help to determine the phenotype (Bernal et al. 2013; Carter and Worthington 2016; Roseman and Delezene 2019). More generally, a trade-off size cascade of the ICM and AD model have been proposed to be a developmental line of least resistance (Young et al. 2015). Whether general or specific, the value of this class of models is that they create a suite of predictions against which observations can be tested and used to learn about both evolutionary rules and their exceptions (Roseman and Delezene 2019).

## Materials and methods

### Materials

To evaluate patterns of variation within extant populations and species, we combined existing documentation of molar ratios and their variance with new measurements. Although there is a rich publication record of population- or species-level statistics on individual molar sizes, those records were not suitable because we could not estimate the variance structure of the ratios of molar sizes from the summary statistics themselves. Instead, we were able to use two types of literature-based data: (1) means and standard deviations (SDs) of molar size ratios for populations or (2) sizes of each of the three molars reported at the individual level for multiple individuals within a species. To be included in analyses of intraspecific variation, either kind of data type needed to be identified to the species level or below. Both one-dimensional length and two-dimensional area measurements were included. Length and area measurements were kept separated throughout the analytical process. Where sex and locality data were published, they were included. Fossilized specimens were removed to avoid circularity in results and the possibility of including over-averaged datasets (Labonne et al. 2012). A review of literature related to the inhibitory cascade recovered six publications that included sufficient data to evaluate intraspecific variation in molar ratios (Labonne et al. 2012; Asahara 2014, 2017; Asahara and Nishioka 2017; Monson et al. 2019; Roseman and Delezene 2019). The resulting combined datasets consisted of 1593 length entries for 47 species (Monson et al. 2019), 706 individual-level (Labonne et al. 2012; Roseman and Delezene 2019), and 72 population-level (Asahara 2014, 2017; Asahara and Nishioka 2017) area measurements for 35 species. The combined datasets are reported in Supplementary Tables S1 and S2.

The two datasets (length and area measurements) derived from the literature had little overlap at the species level and unknown overlap at the individual level because not all datasets included specimen numbers. In order to account for potential differences in results due to measurement dimensionality (length vs. area), we assembled a new dataset of each kind of measurement from identical specimens from a single species. These specimens all consisted of complete, erupted molar rows of the cricetid cotton mouse *P. gossypinus* that were opportunistically collected for another study. Although *P. gossypinus* is part of a different clade from the murid *M. musculus*, the species with which the ICM was originally developed, it is broadly morphologically and ecologically similar to *M. musculus* in terms of being a small-bodied myomorph rodent and a dietary generalist with third molars that are smaller than second molars, which are in turn smaller than first molars (Wolfe and Linzey 1977). *Peromyscus gossypinus* lacks a fourth premolar, unlike other cricetid rodents that do not adhere to predictions of the ICM potentially due to the influence of the deciduous fourth premolar tooth bud on the growth of the first molar (Viriot et al. 2002; Labonne et al. 2012). Based on these characteristics, we considered it reasonable to hypothesize that the molars of *P. gossypinus* would follow the predictions of the ICM. The dataset of *P. gossypinus* contains 70 specimens sampled from four states across the species’ range (Table 1). The geographic sampling was intended to capture first-order variation between mainland phylogeographic groups within the species (Boone et al. 1999; Beckmann 2011). Specimens of both sexes were included and pooled into a single sample because *P. gossypinus* is not significantly sexually dimorphic in size and because sex data were not available for all specimens to test for potential effects of sexual dimorphism directly (Dewsbury et al. 1980).

**Table 1:** Comparison of molar size ratios between samples of *P. gossypinus* from different localities across the range of the species

Sample	*N*	Measurement	Teeth compared	Median ratio value	LA	OK	TN	FL
Louisiana	20	Length	M_3_/M_1_	0.678	—	*1*	*1*	*1*
			M_2_/M_1_	0.801	—	*1*	*1*	*0.486*
		Area	M_3_/M_1_	0.596	—	*1*	*1*	*1*
			M_2_/M_1_	0.817	—	*1*	*0.718*	*1*
Oklahoma	20	Length	M_3_/M_1_	0.664	239	—	*1*	*1*
			M_2_/M_1_	0.803	225	—	*1*	*1*
		Area	M_3_/M_1_	0.604	197	—	*1*	*1*
			M_2_/M_1_	0.809	232	—	*0.114*	*1*
Tennessee	10	Length	M_3_/M_1_	0.665	123	102	—	*1*
			M_2_/M_1_	0.815	81	68	—	*0.076*
		Area	M_3_/M_1_	0.595	115	114	—	*1*
			M_2_/M_1_	0.857	64	47	—	*0.114*
Florida	20	Length	M_3_/M_1_	0.669	208	172	84	—
			M_2_/M_1_	0.789	265	225	156	—
		Area	M_3_/M_1_	0.591	215	209	82	—
			M_2_/M_1_	0.82	234	211	153	—

Upper triangle (italics) contains *P*-values from a pairwise Mann–Whitney U-test with Bonferroni correction. Lower triangle (plain type) contains U values from the same tests.

FL, Florida; LA, Louisiana; *N*, sample size; OK, Oklahoma; TN, Tennessee.

Specimens were scanned using micro-computed tomography (μCT) on a Nikon XTH 225 ST at 9.99–15.74 μm voxel resolution, 100–140 kV voltage, 132–198 uA amperage, and exposure time of 500 ms across 1800 projections. Scans and 3D surfaces are reposited on MorphoSource (https://www.morphosource.org). Specimen numbers and MorphoSource media IDs are listed in Supplementary Table S3. Three-dimensional surfaces of each molar row were created in Avizo 9.1.1 (FEI Visualization Science Group, Berlin), then measured in MeshLab v.2016.12 by two observers. Each measurement was taken in triplicate. Measurement error, including both error within and between observers, was evaluated using an analysis of variance (ANOVA) approach to calculate percent repeatability (Yezerinac et al. 1992; Sokal and Rohlf 1995; Roseman and Delezene 2019). Repeatability was, on average, between 93.1% for length measurements and 95.9% for area measurements. Measurement replicates were subsequently averaged into a single measurement for each variable for downstream analyses.

### Phenotype choice

Two types of measurements have been proposed for comparing relative molar size, each with a slightly different biological interpretation (Kavanagh et al. 2007; Hlusko et al. 2016). The first type, molar crown area, was initially measured in developing teeth and a range of murid rodents, establishing a connection between developmental processes and phenotypic variation between species (Kavanagh et al. 2007). The simplest estimation of crown area takes the product of the maximum mesiodistal length and buccolingual width (Kavanagh et al. 2007; Bernal et al. 2013; Halliday and Goswami 2013; Schroer and Wood 2015; Asahara et al. 2016; Evans et al. 2016; Roseman and Delezene 2019). This metric often overestimates two-dimensional surface area, but the degree to which this difference adds error to the results varies among clades (Hlusko et al. 2002; Kavanagh et al. 2007; Renvoisé et al. 2009; Carter and Worthington 2016). We chose to use the rectangular estimate of crown surface area because it allowed us to incorporate previously published measurements and use a standard metric to compare across a broader taxonomic dataset (Asahara 2014; Roseman and Delezene 2019). Unlike mammals, such as arvicoline rodents, for which the rectangular estimate is significantly problematic, none of the mammalian teeth we measured had high degrees of infolding or emargination along the margin, which increases the likelihood that a rectangular estimate is suitable in this case (Hlusko et al. 2002; Kavanagh et al. 2007).

The second suggested measurement type is the MMC or relative mesiodistal length of each molar crown without the incorporation of buccolingual width (Hlusko et al. 2016; Monson et al. 2019). Specifically, the metric focuses on the first and third molars with the assumption that the second molar occupies one-third of total molar row length (Hlusko et al. 2016). The MMC metric was developed in order to find a phenotypic trait that more directly captured a specific patterning mechanism, or a suite of covarying traits with a shared genetic basis that is relatively free from indirect selective influences through pleiotropy (Hlusko et al. 2016). The exact genetic basis of the MMC and the exact connection between the MMC and the developmental processes of the ICM remain unclear (Hlusko et al. 2016; Monson et al. 2019).

### Phenotype comparison

Comparison of the two metrics, area and length, derived from the same sample may help clarify whether the MMC is simply a more accurate form of the ICM phenotype or whether the two ratio patterns are influenced by related but distinct processes (Hlusko et al. 2016). To test the performance of length and area measurements in the *P. gossypinus* dataset against specific ICM predictions, we used reduced major axis (RMA) regression (Sokal and Rohlf 1995; Kavanagh et al. 2007) and Bayesian Markov Chain Monte Carlo (MCMC) model fitting (Hadfield 2010; Roseman and Delezene 2019). Note that we distinguish the ICM model from the broader AD model space (Polly 2007; Young et al. 2015), and do not test for fit within AD model space because of the limited developmental or modular inferences that can be drawn from patterns that fit the AD model (see Predictions of ICM). We tested the following specific predictions, developed based on previous study of the ICM:

RMA regression between M_2_ and M_3_ size, when both are scaled to M_1_ size, should explain a significant amount of variation within the sample (Kavanagh et al. 2007; Bernal et al. 2013).
The same RMA regression should result in a linear model not significantly different from the equation M3 = 2M_2_ − M_1_ (Kavanagh et al. 2007; Young et al. 2015).
The linear model of the ICM should be able to predict the ratio of M_3_ to M_1_ size based on the ratio of M_2_ to M_1_ size (Kavanagh et al. 2007; Polly 2007) following the equation:
  (1) $$\mu \left(\frac{M_{3}}{M_{1}}\right)=2\mu \left(\frac{M_{2}}{M_{1}}\right)-1$$
The size of M_2_ should be one-third the size of the complete molar row (Kavanagh et al. 2007; Young et al. 2015; Hlusko et al. 2016).
Variance of M_3_ size standardized to the total molar row size should equal the variance of similarly standardized M_1_ size (Young et al. 2015).
Variance–covariance structure of the molar sizes should be predictable through the following three equations (Roseman and Delezene 2019):
  (2) $$\sigma^{2}\left(M_{3}\right)=4\sigma^{2}\left(M_{2}\right)+\sigma^{2}\left(M_{1}\right)-4\sigma (M_{1},M_{2})$$
   
  (3) $$\sigma \left(M_{1},M_{3}\right)=2\sigma \left(M_{1},M_{2}\right)-\sigma^{2}\left(M_{1}\right)$$
   
  (4) $$\sigma \left(M_{2},M_{3}\right)=2\sigma^{2}\left(M_{2}\right)-\sigma \left(M_{1},M_{2}\right)$$

To test Predictions 1 and 2, we used the package *lmodel2* to perform RMA regression and estimate a confidence interval (CI) for the slope and intercept of the model using permutation. To test Predictions 3–6, we fit multiple linear response models using the *MCMCglmm* package (Hadfield 2010). Following previous use of these Bayesian MCMC model fitting (Roseman and Delezene 2019), all traits were modeled as Gaussian, and for all traits, priors were set to be a non-informative inverse Wishart prior for residual covariance with covariances set to zero. Burn-in was set to five hundred thousand iterations of the Markov chain. After discarding the burn-in, we sampled every thousandth iteration 1000 times, for a total of 1000 samples over a period of 1 million iterations (Roseman and Delezene 2019). The 1000 samples form a posterior distribution of estimates of the true molar size, relative sizes, variances, and covariances within *P. gossypinus*. In order to account for measurement error within these models, we multiplied the variance within the resulting posterior sample by the repeatability for the appropriate measurement.

To compare the posterior distribution of observed population values to the theoretical values predicted by the ICM, we used the posterior distribution and mathematical expectations of Predictions 3–6, including Equations (1)–(4) to create theoretical posterior distributions. For example, we applied the posterior distribution of M_2_/M_1_ sizes to Equation (1) to create a distribution of predicted M_3_/M_1_ sizes, then compared that predicted theoretical distribution to the observed distribution of M_3_/M_1_ sizes, then compared the theoretical and observed predictions. To make these comparisons in a standardized system that can be compared with results from other studies, we divided each observed value by its theoretical value, then took the mode and 95% highest posterior density (HPD) intervals for each parameter (Roseman and Delezene 2019). If the 95% HPD interval encompasses one, then we consider the prediction to be consistent with the ICM because there is no difference between the theoretical and observed values. The parameters we analyzed in this way are mean M_3_ size as predicted by the ICM linear predictor (Prediction 3), the mean M_2_ size relative to the total molar row (Prediction 4), variance of M_3_ relative to the total molar row (Prediction 5), variance of M_3_ (Prediction 6), covariance between M_1_ and M_3_ (Prediction 6), and covariance between M_2_ and M_3_ (Prediction 6).

Length and area measurements were evaluated separately for each prediction. If either length or area fit model predictions better, we used that single measurement type in downstream analyses. If both or neither was a good fit, then we continued to analyze both measurements downstream to allow the greatest amount of comparison with past and future studies.

### Levels of standing variation

To describe and compare standing levels of variation in molar proportions, we used the CV (Simpson 1944; Gingerich 1974). CVs are less commonly calculated for ratio values, in part because it is difficult to interpret any comparison between CVs based on different traits with different variational properties (Pearson 1897; Atchley et al. 1976; Lande1977). However, comparison of CVs calculated from the same trait appears to be a reasonable use of the statistic (Lande 1977). In this study, we do not directly compare CVs across different scales (e.g., length vs. area) or between different traits. Factors such as sexual dimorphism and geographic variation can inflate levels of total-species variation and become conflated with other sources of variation (Plavcan and Cope 2001). However, the nature of most of the data used in this study did not allow for testing of whether species-level pooling results in greater variation than sampling sexes or regions separately. There were two exceptions in our dataset, Roseman and Delezene’s (2019) tooth measurements of apes and the tooth measurements of *P. gossypinus* generated for this study.

In the two cases where we could test for potential inflation of variance due to pooling, we used the sign test to investigate whether the CV of each locality or sex subsample was less than the respective CV for the pooled, species-level sample. In order to further determine whether subsamples could be pooled at the species level, we performed a pairwise Mann–Whitney U-test between samples from different states or of different sexes (Asahara 2014). Pairwise tests were corrected using the Bonferroni correction. If populations of *P. gossypinus* were not significantly different from one another, then the sample was pooled at the species level for downstream analysis. If ratio values were significantly different between sexes of primate species, sexes were treated as separate samples. Where different summary values were recorded for different localities, localities were treated as separate populations (Asahara 2014). Where no locality or sex data were reported for samples, a species was pooled into a single sample. Summary statistics for each applicable ratio value for each sample were calculated and are provided in Supplementary Tables S1 and S2.

### Simulating sample sizes

In order to evaluate the minimum sample size necessary to estimate mean and variance structure of molar proportions, we first resampled the *P. gossypinus* dataset without replacement to simulate different sample sizes 2 < *N* < 70. Ten thousand resampled pseudoreplicates were created at each *N*, and mean and variance of each ratio were calculated from each pseudoreplicate. We considered a sample size adequate if 95% of pseudoreplicates produced a mean and variance that fell within the 95% CI of the complete *N* = 70 sample. Based on the results from *P. gossypinus*, we checked to see if any other species in the published datasets had likely been sampled enough to estimate true mean and variance. For those species, we performed the same resampling analysis in order to check for consistency of results across species.

### Simulating use of composite molars

To assess the accuracy of using isolated molars to calculate molar ratios, we used the pooled sample of *P. gossypinus*. To simulate the error resulting from the creation of composite molar rows from averages of isolated teeth, we created 50,000 datasets of simulated samples of isolated teeth. To create each simulated sample, we resampled with replacement *N* teeth of the same tooth position from any of the 70 specimens of *P. gossypinus*, where *N* is the sample size of a single tooth position, ranged from 1 to 50. In order to create a sample-level point estimate of molar size ratios, we took the mean of each of those *N* specimens of each molar position to calculate M_1_, M_2_, and M_3_ crown areas constituting a single composite molar row. For example, a simulation at *N* = 2 would take an average of two “isolated” M_1_s, an average of two “isolated” M_2_s, and an average of two “isolated” M_3_s, then calculate a size ratio of the three means.

To estimate variation for each simulated sample, we further resampled one molar from each tooth position in the simulated sample and used that set of three teeth to calculate ratios. We repeated that process 1000 times to create a “pseudosample” distribution of ratios around the mean value for the composite tooth row, then calculated the SD of that pseudosample. The entire process repeated 999 more times to produce 1000 estimates of the mean and SD of molar ratio values sampled at *N* teeth per tooth position. This process mimics that resampling methods that would be available to paleontologists trying to estimate a CI based on a collection of *N* isolated teeth. We compared those estimates to the 95% CI interval of the true mean and SD of ratios calculated from complete molar rows of *P. gossypinus*. If <5% of the 1000 estimated means for a given *N* fell outside of those CIs, then we considered a composite sample of at least *N* teeth per tooth position to adequately approximate a point estimate of the mean and SD.

All analyses were conducted in R 3.5.3 (R Core Team 2015). All resampling was conducted with 1000 pseudoreplicates (Kowalewski and Novack-Gottshall 2010). Code is reposited on GitHub (project name “molar_ratio_sampling”) with a snapshot archived on FigShare (doi: 10.6084/m9.figshare.12597596).

## Results

**Length or area**

**Fig. 1:**
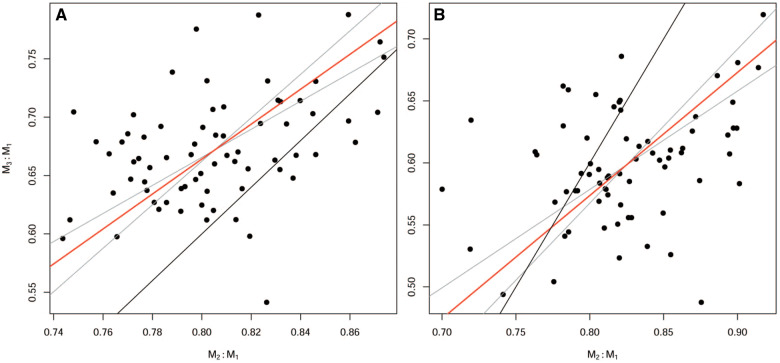
Plot of relative molar size of specimens of *P. gossypinus* in terms of (**A**) length and the MMC; (**B**) area and the ICM. Red line surrounded by gray CIs indicates the best fit RMA regression line explaining variance in the data. Black line indicates the regression line predicted by the MMC and ICM, respectively.

**Fig. 2:**
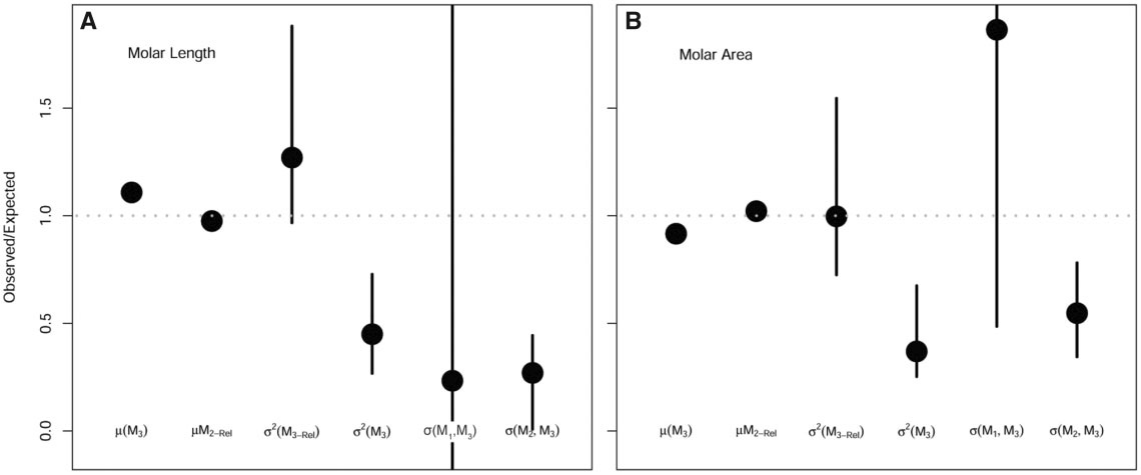
Plot comparing (**A**) MMC and (**B**) ICM predictions to direct estimates of six variables based on a sample of *P. gossypinus* following Roseman and Delezene (2019). The *y* axis measures the proportion of direct estimates fulfilled by predictions. Values higher than one are over-predicted by models. Values less than one are under-predicted. Black points indicate the mode of the posterior distribution and black lines indicate the 95% posterior credible interval. Values on the *x* axis, from left to right, indicate mean M_3_ size (µ[M_3_]), mean size of M_2_ as a proportion of total molar row size (µM_2-Rel_), variance of relative M_3_ size (σ^2^[M_3-Rel_]), variance of the absolute M_3_ size (σ^2^[M_3_]), covariance of M_1_ and M_3_ (σ[M_1_, M_3_]), covariance of M_2_ and M_3_ (σ[M_2_, M_3_]).

Both length and area measurements met few of the predictions of the ICM (Figs. 1 and 2). Although the cluster of values for specimens overlap with the ICM linear model, sample-specific linear models accounted for relatively small amounts of variation between molar ratios (Fig. 1, Table 2). CIs of slopes and intercepts for those sample-specific models did not include values matching the ICM.

**Table 2:** Comparison of molar size ratios between samples of *P. gossypinus* from different localities across the range of the species

	Slope				Intercept			
		95% CI				95% CI		
Measurement	Value	Lower	Upper	Prediction	Value	Lower	Upper	Prediction
Length	1.497	1.203	1.863	2	−0.533	−0.828	−0.297	−1
Area	0.993	0.793	1.242	2	−0.22	−0.426	−0.056	−1

Prediction refers to the values predicted if sample met the expectations of the MMC (length) or ICM (area).

For length measurements of *P. gossypinus*, the only parameter that met ICM expectations was covariance between size of M_1_ and M_3_, although the credible interval is so wide as to make this result meaningless (Fig. 2A, Supplementary Tables S4 and S5). Variances and covariances between raw lengths were lower than predicted by the ICM, but relative variance of M_3_ size was higher than expected. That is, its standardized variance was greater than the standardized variance of M_1_ size. The size of M_3_ was larger than expected, and concordantly the size of the M_2_ in the context of the complete molar row was smaller than expected.

Results for molar crown area measurements were qualitatively similar to length measurements in terms of variance and covariance structure between raw measurements (Fig. 2B). One major difference is that relative variance of M_3_ size met expectations of the ICM. In terms of means of raw and relative size, M_3_ was too small and M_2_ was too large relative to the size of the molar row, opposite the results for length measurements of the same teeth.

### Standing variation in proportions

**Fig. 3:**
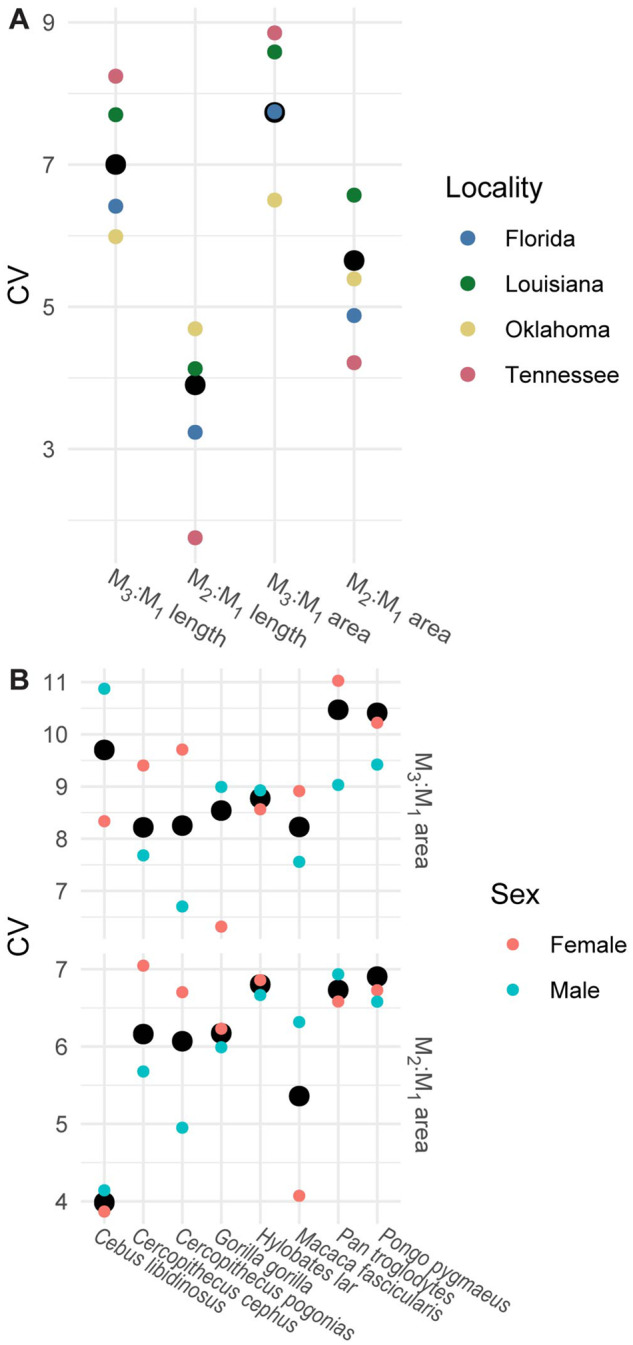
CV of samples of (**A**) *P. gossypinus* from individual localities (small colored circles) or (**B**) species of primate subdivided into sexes (small colored circles) in comparison to a pooled sample (large black circles).

For all ratios, there was no difference in proportions between any of the locality-level samples of *P. gossypinus* (Table 1, *P* > 0.114) and CV of a pooled sample was not consistently higher than those of locality-level samples (Fig. 3A, *P* > 0.5982). The specimens were subsequently pooled into a single sample for further analysis. Primates had significantly different ratio values by sex (Table 3), though CVs of pooled samples were not consistently higher than single-sex samples (Fig. 3B, *P* > 0.298). Primate data from Roseman and Delezene (2019) were separated by sex in downstream analyses.

**Table 3:** Comparison of molar size ratios between sexes of various primate species

	Female		Male			
M_3_:M_1_ area	*N*	Median	*N*	Median	*P*	U
*Cebus libidinosus libidinosus*	30	0.589	34	0.58	0.888	521
*Cercopithecus cephus cephus*	23	1.168	48	1.162	0.869	566
*Cercopithecus pogonias grayi*	27	1.27	32	1.242	0.322	498
*Gorilla gorilla gorilla*	32	1.149	45	1.206	0.005	448
*Hylobates lar carpenteri*	16	1.122	16	1.115	0.539	111
*Macaca fascicularis fascicularis*	28	1.514	33	1.576	0.177	368
*Pan troglodytes troglodytes*	43	1.013	31	1.074	0.041	480
*Pongo pygmaeus*	16	1.015	16	1.116	0.032	71
M_2_:M_1_ area						
*Cebus libidinosus libidinosus*	30	0.884	34	0.879	0.995	511
*Cercopithecus cephus cephus*	23	1.295	48	1.278	0.483	610
*Cercopithecus pogonias grayi*	27	1.377	32	1.345	0.052	560
*Gorilla gorilla gorilla*	32	1.238	45	1.253	0.035	516
*Hylobates lar carpenteri*	16	1.131	16	1.173	0.184	92
*Macaca fascicularis fascicularis*	28	1.387	33	1.356	0.504	509
*Pan troglodytes troglodytes*	43	1.112	31	1.134	0.3	571
*Pongo pygmaeus*	16	1.117	16	1.168	0.08	81

U- and *P*-values come from pairwise Mann–Whitney U-tests with Bonferroni correction.

**Fig. 4:**
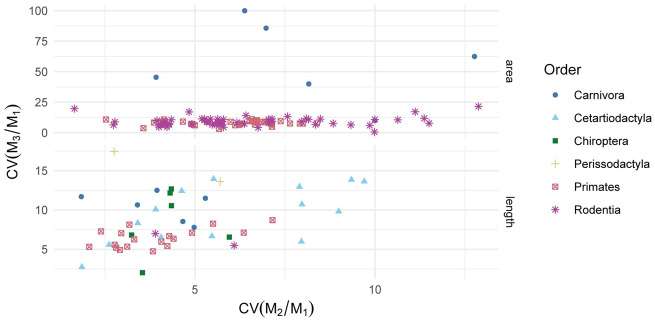
CVs of the ratios calculated to study the inhibitory cascade (top) or MMC (bottom).

Ratios measuring relative M_3_ area were notably high in Carnivora, but these samples all represent populations of the raccoon dog *Nyctereutes procyonoides* in which some ratio values are 0 because the M_3_ is absent (Fig. 4, Supplementary Table S1; Asahara 2014). When samples of *N. procyonoides* are excluded from the dataset, CVs of ratios from area measurements are lower and approximately equivalent to values derived from length measurements and CV was generally similar across species (Fig. 4; Supplementary Table S2).

### Suitable sample size

**Fig. 5:**
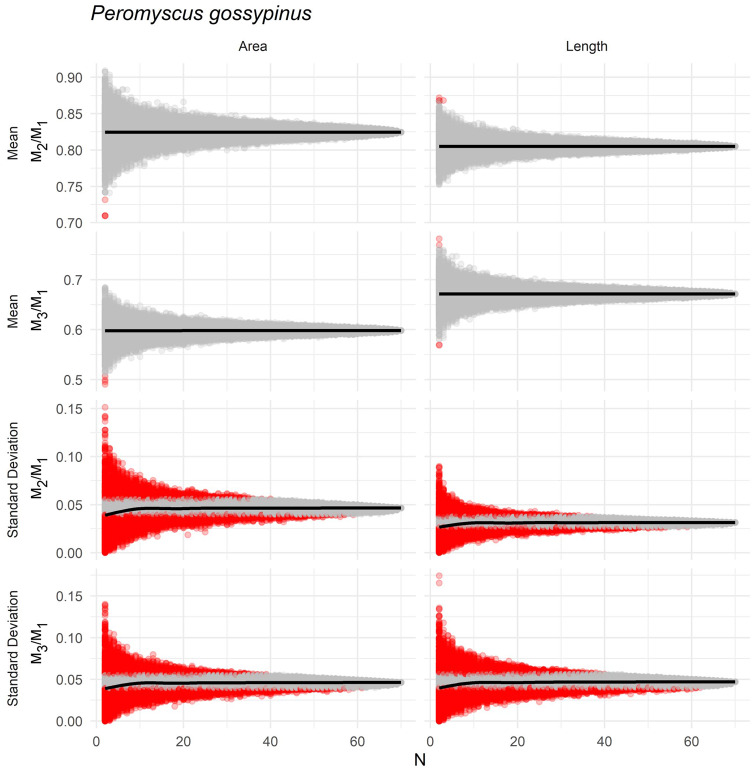
Relationship between sample size and estimates of mean and SD of ratios calculated to study the ICM (area) or MMC (length) based on resampling complete molar rows of *P. gossypinus*. Each gray point is one of 10,000 pseudoreplicates sampled at *N* sample size. Black line indicates the mean value calculated from pseudoreplicates. Red points indicate pseudoreplicate samples whose mean or SD is outside the 95% CI around the observed value calculated for the complete *N* = 70 sample.

Estimates of mean ratios values were less sensitive to sample size than SD of the same ratios in *P. gossypinus* (Fig. 5, Supplementary Table S6). At *N* = 2 specimens or greater, 95% of sample means was within the HPD of the mean of the species for all ratio measurements. SD was more sensitive to sample size, with average SD at low sample sizes (*N* < 3) underestimating the true SD (Fig. 5). Depending on ratio type, at least *N* = 33–42 specimens were necessary to estimate an SD that was likely to fall within the 95% HPD of the true SD.

Eighteen other samples had at least 43 specimens for length measurements and three samples had at least 43 specimens for area measurements. Applying the same analyses to these samples yielded similar results, although five samples required 50 or more individuals before SD were likely to fall within the 95% HPD (Supplementary Table S7).

### Use of composite molar rows

**Fig. 6:**
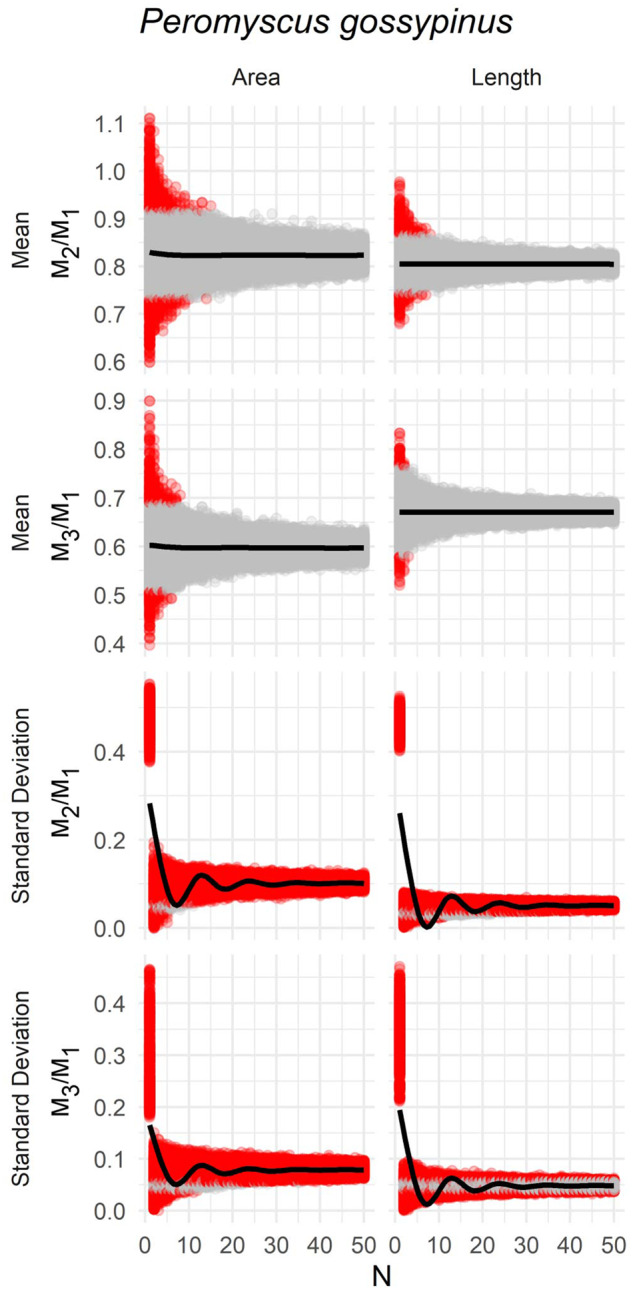
Relationship between sample size and estimates of mean and SD of ratios calculated to study ICM (area) or MMC (length) based on resampling individual molars from molar rows of *P. gossypinus* to make composite molar rows. Each gray point is one of 10,000 pseudoreplicates sampled at *N* sample size. Black line indicates the mean value calculated from pseudoreplicates. Red points indicate pseudoreplicate samples whose mean or SD is outside the 95% CI around the observed value calculated for the complete *N* = 70 sample.

Mean length ratio values calculated from composite molar rows were within the 95% CI of the true mean 95% of the time even at sample size of *N* = 2 (Fig. 6, Table 4). At *N* = 4 or greater, 95% of estimated means was within the 95% CI of the true mean regardless of measurement type. The true variance was not recovered in composite samples regardless of sample size. Estimated variance was frequently greater than the true variance, especially as sample size increased (Fig. 6, Table 4).

**Table 4:** Proportion of samples of composite molar rows that resulted in statistics outside of 95% highest posterior density (HPD)

	Area				Length			
	M_2_/M_1_		M_3_/M_1_		M_2_/M_1_		M_3_/M_1_	
*N*	Mean	SD	Mean	SD	Mean	SD	Mean	SD
1	0.237	1	0.148	1	0.117	1	0.04	1
2	0.108	0.813	0.066	0.787	0.055	0.78	0.005	0.834
3	0.066	0.866	0.034	0.75	0.036	0.738	0	0.705
4	0.05	0.927	0.012	0.755	0.011	0.74	0	0.668
5	0.027	0.959	0.011	0.829	0.009	0.792	0	0.577
6	0.015	0.981	0.007	0.854	0.006	0.835	0	0.521
7	0.008	0.989	0.006	0.884	0.001	0.858	0	0.469
8	0.003	0.992	0.001	0.914	0.001	0.895	0	0.427
9	0.005	0.996	0	0.92	0	0.892	0	0.421
10	0.002	0.999	0	0.945	0	0.911	0	0.399
11	0.001	0.999	0	0.947	0	0.94	0	0.362
12	0.001	1	0	0.964	0	0.947	0	0.309
13	0.003	1	0	0.968	0	0.944	0	0.311
14	0	1	0	0.977	0	0.958	0	0.3
15	0.001	1	0	0.981	0	0.96	0	0.268
16	0	1	0	0.983	0	0.969	0	0.25
17	0	1	0	0.982	0	0.982	0	0.236
18	0	1	0	0.993	0	0.979	0	0.21
19	0	1	0	0.991	0	0.983	0	0.229
20	0	1	0	0.993	0	0.989	0	0.207
21	0	1	0	0.992	0	0.99	0	0.183
22	0	1	0	0.995	0	0.989	0	0.162
23	0	1	0	0.999	0	0.997	0	0.159
24	0	1	0	1	0	0.993	0	0.14
25	0	1	0	0.997	0	0.996	0	0.149
26	0	1	0	0.999	0	0.998	0	0.14
27	0	1	0	0.998	0	0.997	0	0.113
28	0	1	0	0.999	0	0.996	0	0.129
29	0	1	0	1	0	0.997	0	0.111
30	0	1	0	1	0	0.999	0	0.106
31	0	1	0	1	0	0.999	0	0.101
32	0	1	0	1	0	0.998	0	0.096
33	0	1	0	1	0	0.997	0	0.096
34	0	1	0	1	0	0.999	0	0.087
35	0	1	0	0.998	0	1	0	0.064
36	0	1	0	0.999	0	1	0	0.081
37	0	1	0	1	0	0.999	0	0.074
38	0	1	0	1	0	0.999	0	0.075
39	0	1	0	1	0	1	0	0.076
40	0	1	0	1	0	1	0	0.061
41	0	1	0	1	0	0.999	0	0.071
42	0	1	0	1	0	1	0	0.056
43	0	1	0	1	0	1	0	0.065
44	0	1	0	1	0	0.999	0	0.062
45	0	1	0	1	0	1	0	0.058
46	0	1	0	1	0	1	0	0.043
47	0	1	0	1	0	1	0	0.047
48	0	1	0	1	0	1	0	0.042
49	0	1	0	1	0	1	0	0.051
50	0	1	0	1	0	1	0	0.057

*N*, the number of specimens sampled to calculate average size of an individual molar in composite row.

**Fig. 7:**
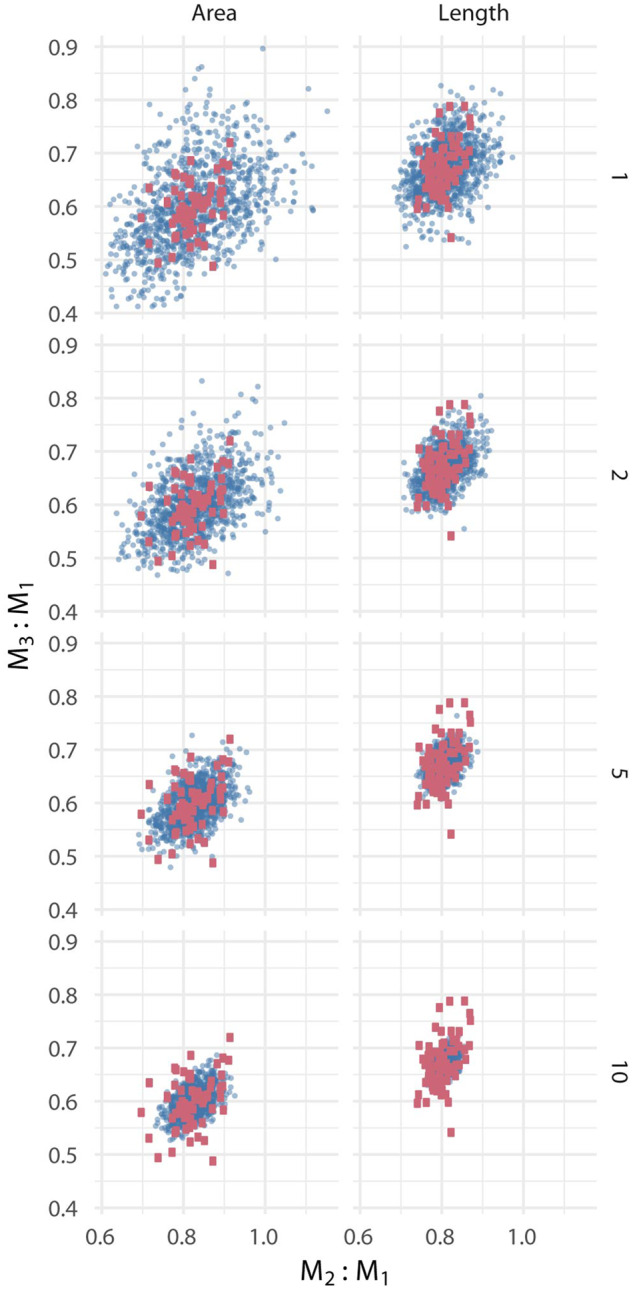
Comparison of occupation of ICM (area) or MMC (length) ratio morphospace by complete versus simulated molar rows derived from the same sample of *P. gossypinus*. Large red squares represent 70 complete molars rows. Small blue circles represent point estimates of ratios calculated by taking the mean of 1, 2, 5, or 10 isolated molars per tooth position per pseudosample.

An alternative way of visualizing these results is to compare the distribution of the composite-based estimates of mean ICM values to the distribution of complete specimens in ratio ICM ratio space (Fig. 7). At *N* = 1, a collection of composite estimates occupies a greater range of morphospace than the true population sample (Fig. 7). At *N* = 5, the composite estimate is already notably reduced to approximately the extent of the true population range, and by *N* = 10 the range of estimates of the mean was more precise than an estimate than many estimates would be based on a single, complete molar row.

## Discussion

Our intraspecific sample of *P. gossypinus* failed to meet the predictions of the ICM, similar to previous findings in primates. The choice of measuring molar size as a length or an area did not affect prediction fit. Overall, the two measurements provided similar results, but differed in details that might lead to different conclusions about why the observed distribution of ratios did not fit the ICM. For example, if we are using tooth crown area (Fig. 2A), we might look for a reason why M_3_ size was smaller than expected, but if using tooth crown length we would look for a cause of an apparently opposite result of M_3_ size larger than expected (Fig. 2B). These results alone do not clarify which measurement is preferable.

Although these ratio phenotypes cannot be fully explained by the ICM, they show promise for future studies addressing the source and history of their covariation. Use of relatively small samples of either complete or composite molar rows is adequate to characterize average molar ratios. Rare or fragmentary records of species, extant or extinct, may be more suitable for study of the molar module than previously considered. Mean ratio values are much less sensitive to sample size than estimates of variance, matching general statistical observations (Gotelli and Ellison 2004).

Assembling datasets with the requisite number of specimens to study variance structure will likely require pooling multiple localities and potentially sexes. Pooling increases the likelihood that additional sources of variation, such as sexual dimorphism and genetic differentiation, may influence results (Albrecht and Miller 1993). For example, some species have documented differences in molar ratio values between populations (Asahara 2014), and these differences may influence estimates of variance structure within the species. To address whether pooling may be the source of increased variability, the standard variability metric CV is often calculated and compared against a standard (Barnosky 1990; Plavcan and Cope 2001). Standing species-level CV for tooth sizes, both in terms of lengths and areas, are already well documented (Wallace 1968; Gingerich and Ryan 1979; Gingerich and Winkler 1979; Sakai 1981; Williamson 2001; Tornow et al. 2006; Natsume et al. 2008). Based on such documentation, an accepted diagnostic principle has emerged that measurements of size of molars themselves are variable within a species up to about CV = 10% or less (Gingerich 1981; Plavcan and Cope 2001). Higher variability is usually interpreted as a sign of over-pooling or the presence of more than one species in the sample (Plavcan and Cope 2001). Variability in ratios of molar sizes is less well understood, and based on results of this study the same recommended standard for molar sizes too low for molar ratio values. Ratios of molar size appear to be more variable than the sizes of the molars themselves, probably because of the way in which error propagates through the calculations.

### Recommendations for future studies

Based on the results of this study, a minimum of 40–50 specimens from a single species is required to accurately study variance structure of relative molar sizes. A far smaller sample, as small as *N* = 2 is adequate for characterizing the mean relative sizes of molars in complete tooth rows. Use of averages of samples of isolated molars to calculate molar ratios within ICM is acceptable with sample sizes of at least four specimens per tooth position, assuming the samples are not overly time-averaged for the question of interest (Kowalewski and Bambach 2008). We consider a CV >15% for either the M_3_:M_1_ or M_2_:M_1_ ratio in a sample of pooled specimens an indicator that the sample may be overly averaging in some way, such as over time, space, or sex. This approach requires a careful choice of how samples are pooled into groups, as well as a consideration of variance in the sample (Kidwell and Holland 2002), but with those caveats the practice allows the ICM to be studied in systems that were previously considered unapproachable. In cases where tooth rows are frequently incomplete, such as in the fossil record, bootstrap resampling of collections of at least five molars per tooth position can produce CIs that are comparable to the natural variability within a species (Fig. 7). Isolated molars are not suitable for estimating variance structure of ratio values.

### Remaining challenges to understanding the ICM

The ICM has been proposed to be a line of developmental least resistance in evolution (Young et al. 2015). Such a line, if comparable, should behave similarly to a genetic line of least resistance, or a linear model of multiple traits that describes the maximum vector of genetic variation within a population (Schluter 1996). Genetic lines of least resistance scale from populations to species, such that patterns of evolution between closely related species bear resemblance to primary patterns of variation within a single species, and statistically speaking the vectors of maximum variance between the two scales of evolution covary (Schluter 1996; Marroig and Cheverud 2010). The behavior of the ICM is unusual from this perspective in that even though the vector of maximum ratio variance between an inbred laboratory strain of *M. musculus* is statistically indistinguishable from a between-species vector of maximum ratio variance in murid rodents, variation documented in this study and others at the intermediate level of standing variation in natural populations does not follow the same vector (Roseman and Delezene 2019).

One necessary test unable to be conducted in this study is whether variation in wild population of *M. musculus* itself, the original subject of ICM studies, follows the ICM. Results of that test would help establish whether the ICM truly does not hold at intermediate biological levels, contrary to the concept of a line of least resistance, or if a wider range of species than previously appreciated do not follow the ICM. Notably, the sample of *P. gossypinus* occupies the same region of ICM morphospace as the ICM linear model, meaning that if the species had been studied in terms of a species-level means it would be consistent with the results of Kavanagh et al. (2007).

The ability to incorporate a greater proportion of the fossil record into studies of relative molar sizes may help explain how certain clades came to be “exceptions” to the ICM. For example, extant equids and ursids not only do not follow the ICM, but also fail to meet the predictions of more general AD models (Polly 2007). Genetic hypotheses have been proposed to explain this deviation in ursids (Asahara et al. 2016) but fossils of extinct stem and crown-ursids may be useful to understand when a shift away from AD model space occurred, what phenotypes the shift is associated with, and what the timing of such shifts can explain about overcoming developmental constraints of patterning mechanisms.

## Supplementary Material

obaa020_Supplementary_Data

## Supplementary data

Supplementary data available at *IOB* online.
